# *“Getting ready for the adult world”:* how adults with spinal muscular atrophy perceive and experience healthcare, transition and well-being

**DOI:** 10.1186/s13023-019-1052-2

**Published:** 2019-04-02

**Authors:** Hamish W. Y. Wan, Kate A. Carey, Arlene D’Silva, Nadine A. Kasparian, Michelle A. Farrar

**Affiliations:** 1 Discipline of Paediatrics, School of Women’s and Children’s Health, UNSW Medicine, UNSW Sydney, Randwick, Australia; 2 Harvard Medical School, Boston, MA USA; 3 Department of Cardiology, Boston Children’s Hospital, Boston, MA USA; 4 Department of Neurology, Sydney Children’s Hospital, Randwick, Australia

**Keywords:** Spinal muscular atrophy, Patient-centered care, Family-centered care, Health services, Lived experience, Quality of life, Mental health, Complex care, Disability, Transition

## Abstract

**Background:**

Spinal muscular atrophy (SMA) has profound implications across a lifetime for people with the condition and their families. Those affected need long-term multidisciplinary medical and supportive care to maintain functional mobility, independence and quality of life. Little is known about how adults with SMA experience healthcare, or the components of care perceived as important in promoting well-being. The purpose of this study was to use qualitative research methodology to explore the lived experiences of healthcare and wellbeing of adults with SMA. Purposive sampling was used to recruit adolescents and adults with SMA, their parents and partners. Face-to-face or telephone-based semi-structured interviews were recorded and analysed using inductive thematic analysis.

**Results:**

Across a total of 25 interviews (19 people with SMA, 5 parents, 1 partner) many participants described disengagement from health services and major gaps in care throughout adulthood. Disengagement was attributed to the perceived low value of care, as well as pragmatic, financial and social barriers to navigating the complex healthcare system and accessing disability services. Adults with SMA valued healthcare services that set collaborative goals, and resources with a positive impact on their quality of life. Mental health care was highlighted as a major unmet need, particularly during times of fear and frustration in response to loss of function, social isolation, stigma, and questions of self-worth. Alongside this, participants reported resilience and pride in their coping approaches, particularly when supported by informal networks of family, friends and peers with SMA.

**Conclusions:**

These findings provide insight into the lived experiences, values and perspectives of adults with SMA and their carers, revealing major, ongoing unmet healthcare needs, despite many realising meaningful and productive lives. Findings indicate the necessity of accessible, patient- and family-centered multidisciplinary care clinics that address currently unmet physical and mental health needs. Understanding the lived experiences of people with SMA, particularly during times of transition, is critical to advancing health policy, practice and research. Future studies are needed to quantify the prevalence, burden and impact of mental health needs whilst also exploring potential supportive and therapeutic strategies.

**Electronic supplementary material:**

The online version of this article (10.1186/s13023-019-1052-2) contains supplementary material, which is available to authorized users.

## Background

Spinal muscular atrophy (SMA) is a progressive neurodegenerative disorder that exerts a significant and complex disease burden. Primary clinical manifestations include progressive symmetrical proximal muscle weakness and wasting, with corresponding losses in motor function [1–5]. There is a broad spectrum of disease severity, and classification is based on age of symptoms onset and motor milestones achieved. SMA type I is the most common subtype, with onset before age 6 months and those affected never sit independently. Children with SMA type II typically develop weakness between ages 6 and 18 months and at peak capacity, are able to sit independently, and often also develop respiratory insufficiency. People with SMA Type III have symptom onset after age 18 months and attain the ability to walk independently, although this ability may not be sustained. Those affected require life-long multidisciplinary medical and supportive care to maintain functional mobility and quality of life [6–8]. Improvements in supportive care coupled with emerging novel therapies are rapidly changing the therapeutic landscape and improving survival; however, research into treatments and healthcare experiences has largely focused on paediatric populations [6, 7, 9–11]. Adults comprise about one-third (35%) of the global SMA population [12], yet there is limited knowledge on how adults with SMA and their families experience this condition from a physical, psychological or social perspective [13–15].

Patient, family and community involvement in health service design, implementation and research [16, 17] is progressively developing and can contribute to improved health system delivery, patient outcomes, and prioritisation of future health policy, practice and research [17–19]. In rare, under-explored populations like adults with SMA, there is a need to develop a deeper understanding of people’s lived experiences to ensure delivery of high-quality, evidence-based, patient- and family-centred healthcare. Qualitative methods are optimally suited for gathering new knowledge and developing a rich understanding of patients’ experiences [20, 21], particularly in terms of how people may construct meaning and derive value from healthcare. While previous research has examined ways in which adults with SMA perceive their quality of life [14, 22–28], no studies have explored how adult SMA patients and their careers experience the healthcare system, nor whether the findings of existing quality of life studies may be transferrable to the Australian context. In Australia, young people with chronic health conditions and disabilities transition from paediatric to adult specialist health care services, often with difficulty if structured and supportive transition programs are not in place [29]. The primary aim of this study was to qualitatively explore the lived experiences and perspectives of adults with SMA, particularly as related to healthcare, transition and wellbeing.

## Methods

### Study design

Qualitative cross-sectional study of adolescents and adults with SMA, and their parents and partners recruited from clinical databases at the Sydney Children’s Hospitals Network (SCHN) and SMA Australia.

### Sample and recruitment strategy

The study aimed to explore the views and experiences of two groups of participants; Group 1 included adolescents and adults aged 14 years or older with a clinical or genetic diagnosis of SMA. Individuals were eligible for participation if they had sufficient English language skills to take part without the aid of an interpreter. As a method of source triangulation, parents and partners of young people with SMA were also approached (Group 2).

The recruitment strategy featured study advertisements distributed via email and social media to members of SMA Australia, the country’s largest SMA patient support organisation consisting of 135 registered adults with SMA. Additionally, invitations were sent via email to potential participants identified using SCHN clinical databases, including patients of the two major referral and clinical management centres for paediatric SMA in the state of New South Wales; Sydney Children’s Hospital and The Children’s Hospital at Westmead.

Due to the rarity of the study population, a broad approach to sampling was initially adopted to garner interest, then purposive sampling was employed to maximise sample diversity (particularly with regards to age, SMA type and gender), to capture the fullest range of perspectives and experiences possible [30]. Informational saturation was defined a priori as the occurrence of no new information emerging from subsequent interviews [31, 32], and we initially estimated this would occur with 20 participants with SMA. The study was approved by the SCHN Human Research Ethics Committee (LNR/17/SCHN/441) and all participants (and, if aged under 18 years, their parents or guardians) provided written informed consent.

### Data collection

A 7-item questionnaire was used to collect clinical and demographic data on age, gender, primary language spoken at home, educational attainment, SMA type, age of symptom onset, and highest level of current physical functioning. Individual semi-structured interviews were carried out by the first author, accompanied by the second author whenever possible, with researcher characteristics detailed in Appendix 1. Interviews were carried out either face-to-face at the Sydney Children’s Hospital or via telephone, based on participant preference, and were recorded then transcribed *verbatim*, with field notes annotated. An interview guide developed after extensive literature review and expert consultation was utilised to facilitate discussion, while the exact wording and sequencing of questions was left open. Opening questions were used to assist in contextualising participants’ experiences of SMA before focusing on issues specific to the adult healthcare experience. These questions encompassed experiences of transition from paediatric to adult healthcare services; perceived strengths, weaknesses and future directions for health services; experiences of the physical, psychological and social consequences of SMA; and strategies used to cope with the burden of disease. Peer debriefing occurred throughout the data collection, coding and analysis process to ensure reflexivity.

### Data analysis

An inductive thematic approach, utilising the conceptual framework of Miles, Huberman and Saldana [21], was adopted for qualitative analysis. The analytic process involved five stages: (1) the reading of interview transcripts and summarisation of key themes to develop a coding list, (2) first cycle coding of interview transcripts within *NVivo12* utilising the coding list generated, (3) second cycle coding to establish data patterns and form connections between codes, (4) use of data visualisation techniques to identify further data patterns, and (5) extraction of salient findings and final synthesis of key themes and subcategories. One researcher (HW) led the coding and analysis process, but all coding and analytic decisions were discussed and reviewed with all researchers in the study team. For demographic and clinical data, descriptive statistics were generated using Microsoft Excel. The consolidated criteria for reporting qualitative research (COREQ) were utilized to report key aspects of the research team, study context and methods, analysis, findings and interpretations (Additional file 1) [33].

## Results

### Participation rate and sample characteristics

**Fig. 1:**
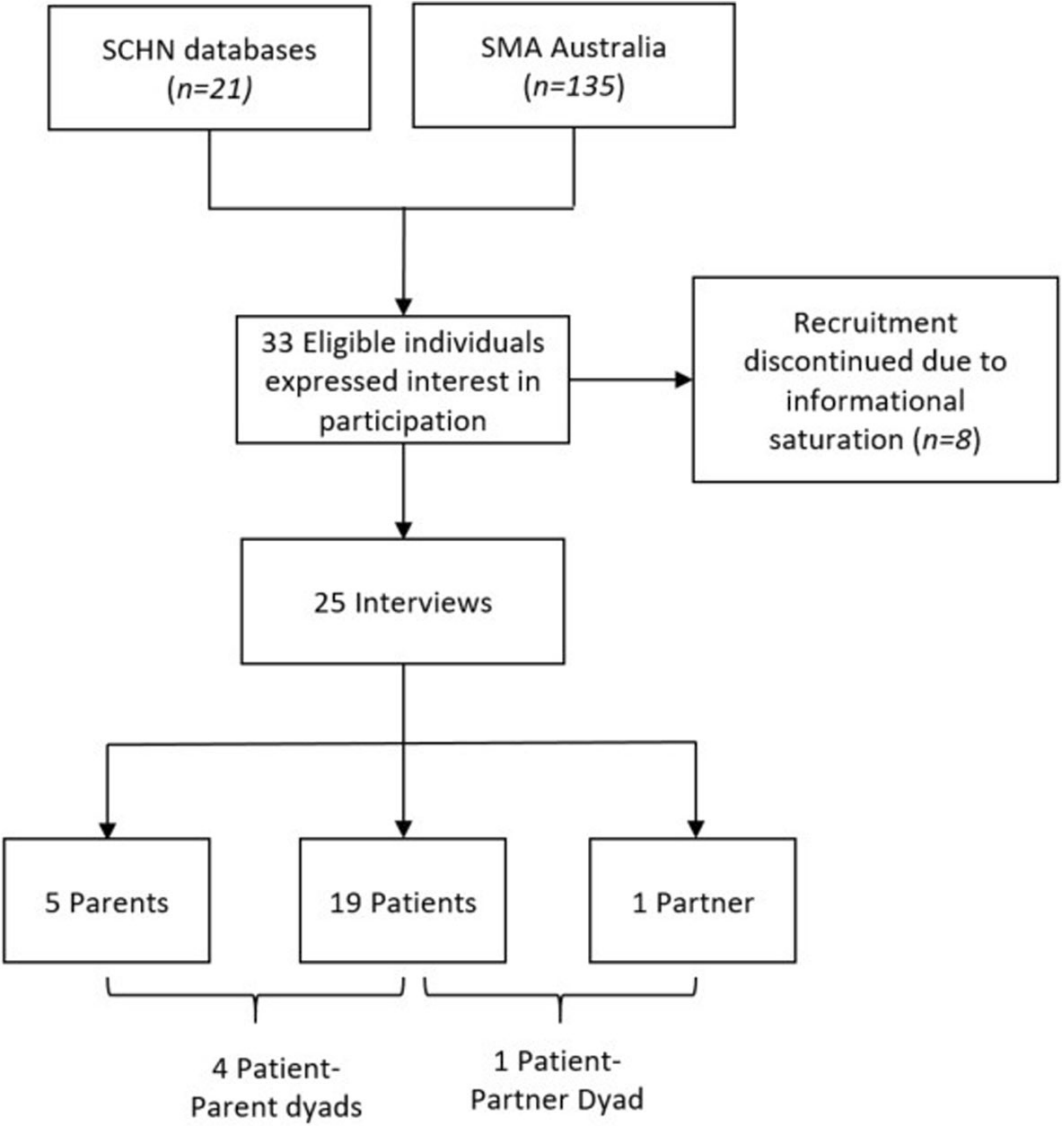
Participant recruitment and data collection. SCHN, Sydney Children’s Hospitals Network; SMA, spinal muscular atrophy

Initially, 156 eligible individuals were identified and invited to take part in the study (see Fig. 1). Of these, 33 individuals expressed an interest in participation and two individuals indicated lack of interest or time and declined to participate. Informational saturation was reached after 19 interviews with people with SMA, supplemented with additional information from five parents and one partner (25 interviews in total, including one patient-partner and four patient-parent dyads who were interviewed separately). Mean interview duration was 55.2 ± 3.3 min (range: 20 to 92 min).

Of those with SMA, 17 had transitioned from paediatric to adult services, one was receiving care in a paediatric setting and 1 was in the process of transition. Sample demographics and clinical characteristics are shown in Table 1 and Appendix 2.

**Table 1:** Demographic and clinical characteristics of the sample (*N* = 25)

Interviewees	People with SMA	Parents^a,b^	Partners^b^
Total number	19	5	1
Age (years)			
Mean (range)	30 (16–48)	57 (49–66)	42
Gender			
Male	7 (37%)	4 (80%)	
Female	12 (63%)	1 (20%)	1
Highest level of education			
Year 10 or below	3 (16%)		
Year 12	3 (16%)	1 (20%)	
TAFE or College	4 (21%)		
Bachelor’s Degree	5 (26%)	4 (80%)	1
Postgraduate Degree	4 (21%)		
SMA type			
I	2 (11%)	1 (16.6%)	
II	8 (42%)	3 (50%)	
III	9 (47%)	2 (33.3%)	1
Highest level of function			
Independent walking	3 (16%)	2 (33.3%)	
Independent sitting	13 (68%)	2 (33.3%)	1
Neither	3 (16%)	2 (33.3%)	
Employment status			
Full-time employment	6 (32%)	4 (67%)	1
Part-time employment	3 (16%)		
Casual employment	1 (5%)		
Full-time student	5 (26%)	1 (17%)	
Retired	1 (5%)		
Unemployed	3 (16%)	1 (17%)	
Primary language spoken at home			
English	16 (84%)	5 (100%)	1
English as a second language	3 (16%)		

^a^One participant had 2 children with SMA (ID003), ^b^“SMA type”, “highest level of function” and “employment status” is relevant for the person with SMA related to them, ^c^SMA classification was based on age of symptom onset and maximal motor function achieved. SMA type I children had symptom onset within 6 months and never attained independent sitting, SMA II had onset between 6 and 18 months and achieved unassisted sitting but not independent walking. SMA III had an onset between 18 months-18 years and attained the ability to walk unaided

### Participant responses and key themes

**Fig. 2:**
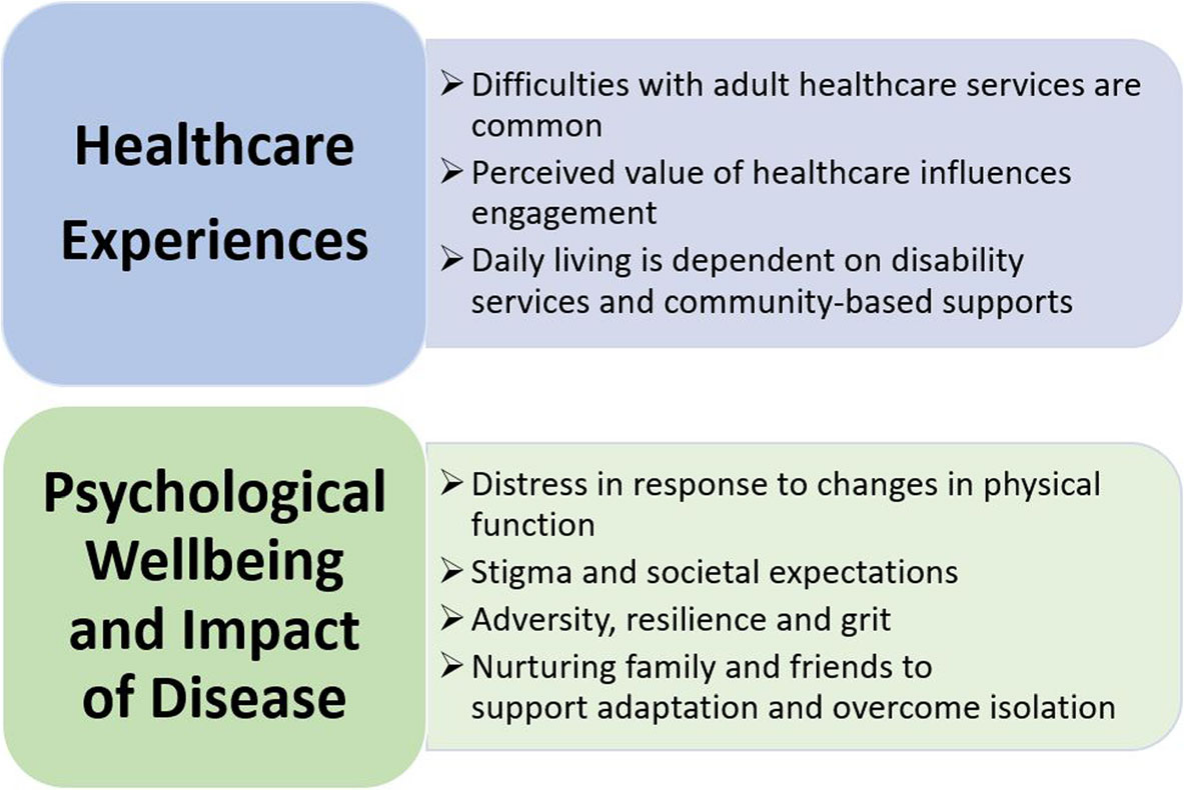
Major themes and sub-themes described by participants about their experiences of living with SMA

Participants’ responses were synthesised into two major themes, “*Healthcare Experiences”* and *“Psychological Wellbeing and Impact of Disease”*, and each theme encompassed several sub-themes (Fig. 2).

### Healthcare experiences

#### Difficulties with adult healthcare services are common

Of the 17 people with SMA who had transitioned from paediatric to adult care, 16 described the experience as *“challenging and scary”.* Difficulties were associated with learning to navigate a new and complex healthcare system and engaging with unfamiliar specialists whose approach was experienced as impersonal and compartmentalising. Many participants described differences in information provision and expectations, with a stronger emphasis on self-management and care co-ordination; *“Going from a children’s hospital where they explain everything in great detail, to the adult [healthcare environment] where they expect you to know the majority of things, you pretty much had to be on the ball all the time.” (ID005, Person with SMA-II).*

Almost all participants reported difficulties identifying and accessing specialists and multidisciplinary clinics for adults with SMA, or perceived available services as inadequate; *“Once you hit the adult system, there’s no one that really seems to know what to do” (ID017, Person with SMA-I).* Participants mostly accessed or were directed to generic healthcare services, where they were required to explain their condition to health providers; “*I still do actually see someone … all he does is check up on me and refer me to other services if I need to. But my experience then is he has no idea really what SMA is” (ID006, Person with SMA-II).* Participants described similar experiences with community-based services; *“Sometimes they send you a carer who has no idea how to use the hoist” (ID022, Person with SMA-III).*

Participants also described widespread difficulties accessing funding and equipment, as well as major resource gaps and a clear lack of support in navigating the system; *“Was I given a lot of assistance and guidance? No. One of the frustrating things was that I knew nothing about the system. I had to sort of stop by a lot of misadventure and difficulty finding out things” (ID003, Mother of SMA-II/III).*

Negative experiences were not universal. Four of 17 adults with SMA described ‘*seamless’* engagement with healthcare providers, occurring in the context of structured, well-supported transitions to adult healthcare. In some cases, successful transition was also attributed to having a consistent healthcare provider throughout the process – a clinician who was well-acquainted with them and their needs; *“He’s [GP] been seeing me since I was pretty young, for about 21 years … he obviously knows me quite well and knows the challenges I have” (ID013, Person with SMA-II).* A strong therapeutic alliance was highly valued; “*It’s pretty important to me that I’ve got a good relationship with my doctor … I have to build up a rapport before I open up to them” (ID008, Person with SMA-II).*

Many participants voiced a desire for tailored, disease-specific, adult multidisciplinary clinics with a strong focus on holistic, patient-centred care co-ordination. Such clinics would also provide access to emerging therapeutic research and clinical trials; *“There needs to be a specialist neurology clinic that deals with people with SMA, that understands all the issues that we have and that can effectively support people to connect with the appropriate allied health supports … the latest research, the clinical trials” (ID017, Person with SMA-I).*

#### Perceived value of healthcare influences engagement

A perceived lack of value in healthcare strongly influenced disengagement. Ten participants described experiencing brief or superficial interactions with disinterested and unknowledgeable healthcare providers, as well as limited access to support, generating a perception that adults with SMA were not valued by the health system; *“That kind of attitude is very, very reflective of what it’s like to be an adult especially with a condition that is degenerative and changes over time … they just don’t really see the value in supporting you physically to help you with better health outcomes” (ID012, Person with SMA-III).* In response, 10 of the 17 adults with SMA described disengaging from doctors or health services following the transition out of paediatric care; *“Anything that you needed from them, even if it was a bare minimum necessity, was difficult to get from the adult services … you would just avoid them unless you had to talk to them, because a lot of the time you just wouldn’t get a lot out of them” (ID012, Person with SMA-III)*. Some disengaged from recommended follow-up after attending a single appointment in the adult healthcare system, often due to a perception of futility and the need for self-protection; *“No one’s going to tell you [anything] good, so why go?” (ID002, Person with SMA-III).*

Variation in disease course also influenced engagement with medical professionals. Most participants with stable symptoms or less severe phenotypes (3/8 adults with SMA-II, 7/9 adults with SMA-III) disengaged from adult health services; *“I felt like it was more of a functional problem, the disease wasn’t changing from year to year and I didn’t really have any questions, so I just didn’t see much point in seeing a neurologist about it” (ID024, Person with SMA-III).*

Conversely, disease progression and functional deterioration prompted adults with SMA to re-assess the potential value of healthcare and actively seek services; *“Then as I got older, my condition just got worse and I felt like I needed to see somebody to see if there was anything they could do to improve my condition at all” (ID013, Person with SMA-II).* The possibility of accessing novel treatments, like nusinersen (Spinraza, the first approved disease-modifying therapy for SMA), was a strong motivator for re-engaging with medical care. Five of the 10 participants not accessing regular medical care reported attempts to re-engage with neurology services soon after nusinersen became available in Australia; *“Only last week I looked [up] the neurologist for adults … if it wasn’t [for] Spinraza, I probably wouldn’t bother” (ID004, Person with SMA-III*). Several participants did, however, express a need for greater patient-physician collaboration; “*Professionals need to understand that people with SMA are the experts on SMA … because no one knows more about SMA than people that live it day to day.” (ID017, Person with SMA-I).*

#### Daily living is dependent on disability services and community-based supports

Reliance on others for care, support and assistance with basic daily needs was perceived as vital but also as a persistent challenge, especially for participants with greater functional impairment; *“I have a home carer who comes and assists me … they help me with showers and stuff, but mum still helps me with my meals and my personal care, like toileting.” (ID007, Person with SMA-III).* Funding and equipment were also perceived as vital; *“I was pretty well bed-bound because if I haven’t got my wheelchair, I’ve got nowhere else to go” (ID023, Person with SMA-II).* Several participants acknowledged inherent disadvantages, mostly practical, when relying on external carers; *“Then you’re in timeframes, so your life is very restricted and you’re in this battle of when my next carer’s coming, I’ve got to do this within this amount of time” (ID023, Person with SMA-II).* In this context of high dependency, continuity of care with trusted carers was perceived as important for maintaining privacy, dignity and comfort; *“I like my privacy and having a thousand different care workers is not my ideal either” (ID007, Person with SMA-III).*

Large gaps in formal care were often filled by informal, unpaid support from family, friends and peers; *“I do often worry about, you know, what would happen if I didn’t have, you know, like, family and that support because there’s - when your care needs are 24/7, you know, the disability support system doesn’t always cater for that. Particularly if you want to live in the community” (ID017, Person with SMA-I).* Practical support provided by family and friends was, however, recognised as unsustainable over time; *“As [my mum] got older, things got harder for her too. She couldn’t lift me to the bed or things like that anymore … and that’s when basically we were recommended to get carers” (ID013, Person with SMA-II).* For some parents, accepting this inevitability was difficult, often due to a desire to protect their child with SMA; *“With my mum and not wanting people doing my personal care, [she] knows how people live alone successfully, but no matter how many times they tell her or encourage her to let go of those ropes, she still won’t” (ID006, Person with SMA-II).*

### Psychological wellbeing and impact of disease

Many participants perceived mental and physical healthcare needs as equally important; “*If you don’t have that mental state, no matter whether you can walk or not, it doesn’t make a difference. You will not be able to communicate with people” (ID016, Person with SMA-II).*

#### Distress in response to changes in physical function

Participants described deep sadness in response to the loss of independence associated with declines in functional capacity; *“When I have to fully rely on someone, that’s when I can see sort of, yeah, depression kicking in - when I have to ask someone to say, brush my teeth or wipe my bum or something like that” (ID018, Person with SMA-III).* This was accentuated by the constant fear of further functional decline; *“I think there is a process of grieving the loss of certain abilities. Then you sort of adapt and accept it and before you know it, there’s something else that you’re then experiencing the loss of” (ID017, Person with SMA-I)*. For some, denial was evident and served to support self-preservation amid intense fear of loss of function; *“I thought that if I read [that] most SMA Type 2s, for instance, lose function by this age, that would become a self-fulfilling prophecy for me … I thought, ‘Well, I don’t want those diagnoses in the back of my mind. I’ll stay away from that and live how I want to live!’” (ID010, Person with SMA-II).* Outward coping at times masked deeper feelings of sadness and loneliness; *“ … well maybe I just put a front up and block it out a lot of the time … maybe I’m … not so resilient” (ID002, Person with SMA-III).* Parents and partners also acknowledged their loved one’s use of denial to cope; *“[My son] has lots of things going on physically that he just [pretends] doesn’t happen … He doesn’t look after himself at all” (ID011, Mother of SMA-III).*

Participants conveyed the importance of recognising both the light and shade of people’s emotional experiences and wanted to be valued, not pitied by others; “*… you shouldn’t assume because someone has SMA they are going to be sad and depressed because it’s coming from a judgement of, you know, that you should be, but that’s not always the case. [My son] recognises when people are pitying him and it frustrates him” (ID009, Mother of SMA-I).*

#### Stigma and societal expectations

Stigma influenced participants’ lived experience of SMA; *“I sort of prescribe to the notion that most of the symptoms of my disability are societal not medical” (ID010, Person with SMA-II).* For some, cultural stereotypes of disability conflicted with what people with SMA could and wanted to achieve; *“Growing up, I was kind of told that when you have a disability, people don’t really want you … We all [siblings] went to uni, but in terms of getting a job, having a family, having a house - just sort of things that people do in life - [my parents] sort of had that attitude where that’s not really going to happen for me” (ID006, Person with SMA-II).* For both men and women, not being able to fulfil certain gender stereotypes led to questions of self-worth; *“I’ve struggled sometimes to deal with self-worth, especially I guess being a female and having those expectations of what a woman should be able to provide in terms of like relationships, umm, and like motherhood*” *(ID006, Person with SMA-II).*

#### Adversity, resilience and grit

Despite health and healthcare challenges, many participants described flourishing through a mixture of personal resilience and informal support networks. This included cultivating a sense of self-worth despite physical limitations, focusing on one’s abilities rather than disabilities, and not comparing oneself to others; “*I think it’s very easy to compare yourself to what other people can do and I try not to do that. I try to focus on what I can do” (ID007, Person with SMA-III).* Overcoming one’s own negative perceptions of equipment (e.g., wheelchairs and hoists), was vital to transcending or adapting to physical limitations; *“The best thing that ever happened was me getting a wheelchair. At the time it was the most depressing thing ever, it was like me giving up, but then I realised the freedom that it had given me to do so many more things” (ID018, Person with SMA-III).*

Self-worth, value and meaning were nurtured by seeking challenges and realising achievements; *“It makes you feel, it gives you a sense of worth as well, that people you know, need you” (ID018, Person with SMA-III).* Educational achievement was seen as a way of empowering people with SMA to dismantle negative stereotypes of disability; *“Personally I feel like I was a high achiever … and maybe now in reflecting … maybe it’s a way of trying to overcompensate for having a disability, like really wanting to prove to myself that I am an equal or if not even better” (ID006, Person with SMA-II).* For others, sustaining social and romantic relationships, or finding joy and pride in raising one’s children were highly valued; “*Being a single mum and doing that singlehandedly, I think that’s my biggest achievement” (ID015, Person with SMA-III).*

Vocational and recreational pursuits were also valued achievements; *“So that’s my biggest achievement - travelling and working for 7 years at my job” (ID004, Person with SMA-III)*. Fatigue and the burden of physical care prevented many participants with SMA from securing full-time employment; however, working from home and flexible or part-time working arrangements facilitated participation in the labour force (see Table 2). Active employment (*n* = 10) or pursuit of full-time studies (*n* = 5) was reported by most people with SMA, covering a diversity of professions (Appendix 1). Six people with SMA perceived workplace discrimination or lack of adequate support in the workplace as barriers to employment; “*To try and find a job which you can do … as soon as they heard you need support, they just wouldn’t employ you*” *(ID005, Person with SMA-II).*

Fostering a sense of independence was also perceived as integral to positive coping; “*Because I was in my 20’s and me and my mum were constantly fighting, you know, it’s a big deal when you’re in your 20’s and your mum still has to shower you … So, I just woke up one morning and said, ‘I’m ringing an agency!’ I just needed to be a bit more independent”* (*ID004, Person with SMA-III).* As an adult with SMA, developing autonomy alongside physical dependence was described as, *“being able to define for yourself how you live your life and also that you are the one that knows what’s best for you. It doesn’t mean you do things yourself, it means you’re the one who decides what gets done, and how it’s done, and you know, who does it” (ID017, Person with SMA-I).*

#### Nurturing family and friends to support adaptation and overcome isolation

Strong, reliable networks of family and friends also supported psychological adaptation; *“Well, I never personally had an issue with [mental health], but I do know people that have, mainly just because of isolation or bullying. But I had a really good circle of friends and a really good family, and I never let that sort of stuff affect me” (ID014, Person with SMA-III).* Social support helped cultivate a sense of normalcy and acceptance; *“To realise that people are there to help you, that was the biggest other hurdle. I used to think I was a hindrance to people” (ID018, Person with SMA-III).*

Support from other adults with SMA provided comfort and inspiration; *“Having someone a lot older to be sort of a peer mentor has really helped me to realise what’s possible … just like knowing, these are the types of jobs and things that they’ve done” (ID006, Person with SMA-II)*. Peers also offered unique practical advice; “*Peer support is really important so that you’re learning from others about the sort of things that you need to know … what services are helpful” (ID017- Person with SMA-I).* There was a sense of camaraderie with others with SMA and a shared understanding of the struggles experienced; “*I think there’s merit in getting assistance through social workers and whatnot, but I don’t think it holds as much merit as seeking it from someone who has actually done it and lived it and experienced it … they know what I’m going through now, so there’s more of an element of trust” (ID010, Person with SMA-II).*

Some participants with SMA experienced increasing social isolation due to physical limitations; “*When I was 25 I wasn’t able to drive anymore. So that made me like, more isolated. Not being able to visit people at their homes. Because I can’t get up off chairs and stuff. Then I started to use my wheelchair. Then you know - I can’t get into people’s houses” (ID014, Person with SMA-III).* With experiences such as these came loneliness and a yearning for social inclusion and a sense of normalcy; *“I think mainly loneliness and isolation are probably the two biggest things … wanting all those normal relationships that others would have without actually having to think about how to actually arrange it” (ID023, Person with SMA-II).* Despite these barriers, 17 of 19 participants with SMA described having established strong social connections and support networks with friends and peers.

## Discussion

Few studies have explored the lived experiences of adults with SMA [22, 23, 25, 26], and to our knowledge, this is the first Australian study. Our findings suggest the care needs of adults with SMA are largely unmet. Participants reported difficulties navigating the health system and accessing health services and perceived the healthcare they did access to be of low value. This, coupled with periods of disease stability, culminated in high rates of disengagement from recommended medical care. In contrast, mental health was perceived as integral to maintaining wellbeing, yet timely access to appropriate mental health services was also highlighted as an unmet need. Over time, participants described cultivating resilience and a strong sense of pride and self-worth; however, many had also experienced periods of intense sadness, fear, frustration and loneliness, particularly in response to physical deterioration and functional decline. These findings provide insight into people’s lived experiences of SMA, as well as evidence to inform improvements in health policy and practice.

### Recognising and addressing the unmet healthcare needs of adults with SMA

The lack of best practice models of care for adults with SMA [6] was reflected in participants’ experiences of inconsistent healthcare quality. This is a problem common across many rare diseases [34], often due to limited knowledge of disease pathophysiology and optimal treatment modalities [35–37]. Limited access to experienced clinicians and professional carers was met by participants with disappointment and frustration, generating a lack of trust and an unwillingness to maintain seemingly unfruitful clinical interactions. This may serve as a catalyst for patients and caregivers to become more self-reliant and to develop innovative strategies to improve their quality of life [38], as previously found for people with other rare diseases, like Von Hippel-Lindau disease [39]. Our participants also strongly affirmed the benefits of peer networks and community-based organisations as a source of information and support [40].

Health behaviours may be influenced by a range of physical, psychological and social factors, including perceptions of risk and disease severity, emotional responses to illness, beliefs about the benefits of and barriers to health actions and social and cultural norms [41, 42]. In our study, participants identified numerous barriers to health service engagement, including fear, denial of disease progression, mistrust of health providers, low perceived value of healthcare, and physical, practical and financial restrictions. Stigma was also widely acknowledged and is recognised in the literature (particularly in disability and mental health), as a major barrier to help-seeking, with people often hoping to avoid appearing “sick” or “weak” [43–45]. We found it often took significant declines in muscle strength and functional ability to motivate re-engagement with healthcare, alongside strong hopes for access to new drug treatments, such as nusinersen [14, 23, 46]. As a first step to addressing the challenge of healthcare disengagement, adult-focused clinical guidelines are needed to define best practices and facilitate standardised care. Participants consistently emphasised the importance of incorporating patient voices into clinical care, service development and quality improvement initiatives, acknowledging the shared wisdom of those with SMA. Experience-based co-design, featuring early, active and ongoing engagement with patients, carers and other key stakeholders, is considered integral to health service quality, producing health services that are better attuned to the issues that matter most to patients. Studies employing this approach describe improvements across a range of outcomes for both patients and health services, including enhanced patient experiences of care, adherence to treatment, use of resources, operational efficiency and reduced formal complaints [47–51]. Health service co-design may be particularly powerful in the rare diseases setting, where clinicians often have limited knowledge and lack disease-specific expertise [34].

Despite major challenges, a small subset of participants described positive healthcare experiences facilitated by access to a formal, structured transition process. In other chronic diseases, such as diabetes, evaluation of different models of transition has shown that structured transition services are associated with higher clinic attendance rates, lower complication rates [52], and greater disease-related knowledge and self-management [53–55]. Optimal transition is thought to be progressive, with discussions and planning initiated when patients are about 14 years of age, depending on developmental capacities [56], and includes dedicated co-ordinators, nurses or social workers to support continuity of multidisciplinary care across the transition period, in partnership with the patient. In a recent survey of 810 Australian adults living with a rare disease, 55% of participants reported problems transitioning from paediatric to adult health services [29]. Our findings suggest that the proportion of SMA patients who encounter difficulty could be even higher and that provision of accessible, organised and evidence-based transition services for young people with SMA could make a meaningful difference to their experience of healthcare.

### Mental health is as important as physical health

From a psychological perspective, previous research has described the distress adults with SMA often experience in response to progressive declines in functional ability and independence, and the threat of death [13, 22, 23, 26, 27, 57]. Extending this knowledge, our study is one of the first to provide an in-depth exploration of patients’ emotional experiences and coping responses. Participants described resilience in adapting to their circumstances, yet outward appearances of coping at times masked more complex and distressing emotions, such as sadness, loneliness, fear and frustration. Fear of physical deterioration and functional decline can have important clinical implications; influencing, for example, engagement with or avoidance of health care. In the oncology setting, fear of cancer recurrence is widely acknowledged to interfere with health behaviours. For some, the fear of illness may drive unnecessary or excessive engagement with health services while for others, fear may serve to delay seeking care when needed [58, 59]. In our study, carers reported worries that their loved one with SMA at times underestimated their symptoms or tried to ignore indications of declining function. Conveying a deeper empathy for our patients’ fears may help to reduce the likelihood of disengagement from health care, particularly during periods of heightened vulnerability, such as the transition from paediatric to adult services. A recent study carried out in The Netherlands found greater symptoms of depression (but not anxiety) were associated with lower participation in and satisfaction with SMA care [57]. The presence of emotional distress has also been shown to be a determinant of health related quality of life in adults with SMA [27]. Overall, there is a need for greater emphasis on the mental health of people with SMA, particularly as studies in other settings have demonstrated the efficacy of integrating tailored mental health services and interventions into existing models of care [60–62].

### Strengths and limitations

The clinical and demographic diversity of our sample is a key strength, with representation of men and women of various ages and from diverse educational, vocational, geographic and cultural backgrounds, as well as people with a range of SMA types and current levels of function. Involving researchers from different disciplines and backgrounds in the process of qualitative data analysis also helped to minimise potential researcher bias. In addition, the primary interviewer had no previous contact with participants. There are, however, several limitations to consider when interpreting the study results. With approximately half the sample recruited via SMA Australia, our participants may represent a more positive, proactive subset of the adult SMA population; however, given the range of negative views and experiences described throughout the study, this seems unlikely. Use of an ‘opt-in’ recruitment strategy may have resulted in less motivated people not taking part and representation of parents and partners of people with SMA was low, potentially limiting the generalisability of findings for these groups. Additionally, as health systems vary internationally, the external validity of our results may be limited; however, many of the raised issues are likely to be experienced by those with SMA worldwide and of broad interest.

### Implications and future directions

The findings of this research have several implications for future policy and practice. First, the results highlight a need for developing and testing well-structured, co-ordinated and patient-centred transition programs tailored to address the needs of SMA patients and their caregivers. While standalone SMA clinics may not be feasible given the rarity of the disease, targeted adaptations to existing chronic care models should be considered. Second, the data highlights major gaps in our understanding of, and response to, the mental health needs of adults with SMA. Future research is needed to provide estimates of the prevalence, predictors, burden and costs associated with psychological distress in this population, as well as to develop and trial effective and sustainable psychological care services, interventions and resources. In addition, continued and creative efforts are needed to reduce the stigma associated with disability and to bolster community-based, peer-led support networks for people affected by SMA and other rare diseases. These new initiatives, including both physician and community education, are likely to benefit from adopting an evidence-based co-design approach, to ensure the expert patient voice is at the centre.

## Conclusion

This study sought to explore the healthcare experiences and perspectives of Australian adults with SMA. Our findings show that the majority of adults disengage from healthcare at the time of transition from paediatric to adult services, often due to the perceived futility or low value of care, as well as psychological, financial and pragmatic barriers to navigating the complex healthcare system and disability services. Adults with SMA report widespread unmet physical and mental health care needs, alongside pride in resilience, personal accomplishments and treasured social relationships. The results suggest greater efforts are needed to develop multidisciplinary, patient-centred health services with well-defined care pathways to support successful transition and care across the developmental spectrum, and to reduce loss to follow-up and disease burden.

### Additional file

Additional file 1: COREQ checklist (PDF 354 kb)

## Appendix 1

**Table 2:** Researcher characteristics

Member (Credentials)	Occupation at time of study	Gender	Experience and training	Relationship to interviewees
HWYW (Undertaking - BMed MD (Hons))	Medical Student	Male	Qualitative research workshop	No prior relationship to interviewees. Introduced to topic by supervisor (MF)
KC (PhD)	Research scientist	Female	10 years experience in scientific research	Previous interactions with some participants due to other research studies.
AD (MPhil)	Research Scientist	Female	5 years experience in scientific research, first qualitative research study	No prior relationship to interviewees. Introduced to topic by supervisor (MF)
NK (BA, PhD, MAPS)	Associate Professor (UNSW), Head of Psychology (Heart Centre for Children)	Female	15 years experience in clinical and research psychology	No prior relationship to interviewees.
MF (MBBS (Hons), FRACP, PhD)	Paediatric Neurologist and Associate Professor (UNSW)	Female	10 years experience in research, 15 years experience in child neurology	Previous interactions with some participants due to other research studies. Paediatric neurologist for 1 participant > 5 years ago prior to transition

## Appendix 2

**Table 3:** Demographical Characteristics

Participant code	SMA Type/ type of participant	Best level of function	Age of symptom onset	Gender	Age (years)	Primary language spoken at home	Highest level of education	Current occupation of person with SMA
ID001	SMA I/ Person with SMA	Neither	6 months	Male	16	English	Year 10 or below	High School Student
ID002	SMA III/ Person with SMA	Able to walk independently	9 years	Female	33	English	Bachelor’s degree	Teacher
ID003	SMA II and III/ Mother	Neither (SMA II), Able to walk independently (SMA III)	< 18 months (SMA II), 9 years (SMA III)	Female	63	English	Bachelor’s degree	Senior Manager
ID004	SMA III/ Person with SMA	Able to sit independently	13 months	Female	27	English	Year 10 or below	Receptionist
ID005	SMA II/ Person with SMA	Able to sit independently	14 months	Female	38	English	TAFE/ College certificate	Unemployed
ID006	SMA II/ Person with SMA	Able to sit independently	9 months	Female	26	Chinese (dialect)	Postgraduate university qualification	Event facilitator
ID007	SMA III/ Person with SMA	Able to sit independently	18 months	Female	27	English	Year 10 or below	Administrator and customer service
ID008	SMA II/ Person with SMA	Able to sit independently	18 months	Female	23	English	Bachelor’s degree	Journalist
ID009	SMA I/ Mother	“Neither”	6 months	Male	49	English	Year 10 or below	High School Student
ID010	SMA II/ Person with SMA	Able to sit independently	18 months	Male	27	English	Postgraduate university qualification	Content producer
ID011	SMA III/ Mother	Able to walk independently	2 years	Female	50	English	Year 12	Car Salesman
ID012	SMA III/ Person with SMA	Able to sit independently	18 months	Female	28	English	Bachelor’s degree	University Student (PhD)
ID013	SMA II/ Person with SMA	Able to sit independently	10 months	Male	34	English	TAFE/ College certificate	Retired (Former IT service desk worker)
ID014	SMA III/ Person with SMA	Able to walk independently	3 years	Female	40	English	TAFE/ College certificate	Doll maker
ID015	SMA III/ Person with SMA	Able to sit independently	3 years	Female	48	English	TAFE/ College certificate	Unemployed
ID016	SMA II/ Person with SMA	Able to sit independently	15 months	Male	24	English	Postgraduate university qualification	Unemployed
ID017	SMA I/ Person with SMA	“Neither”	6 months	Male	42	English	Postgraduate university qualification	Senior policy manager + Adjunct Senior Research Fellow
ID018	SMA III/ Person with SMA	Able to walk independently	1 year	Male	44	English	Bachelor’s degree	National Chief Scientist
ID019	SMA II/ Mother	Able to sit independently	15 months	Female	58	English	Bachelor’s degree	Unemployed
ID020	SMA II/ Person with SMA	“Neither”	18 months	Female	22	Dari	Year 12	University student (Bachelors)
ID021	SMA II/ Father	Able to sit independently	18 months	Male	66	English	Bachelor’s degree	Journalist
ID022	SMA III/ Person with SMA	Able to sit independently	6 years	Female	19	Dari	Year 12	University student (Bachelors)
ID023	SMA II/ Person with SMA	Able to sit independently	< 18 months	Female	28	English	Bachelor’s degree	Consultant
ID024	SMA III/ Person with SMA	Able to sit independently	3 years	Male	24	English	Year 12	University student (Bachelors)
ID025	SMA III/ Wife	Able to sit independently	1 year	Female	42	English	Bachelor’s degree	National Chief Scientist

## References

[1] Farrar MA, Vucic S, Johnston HM, du Sart D, Kiernan MC (2013). Pathophysiological insights derived by natural history and motor function of spinal muscular atrophy. J Pediatr.

[2] Farrar MA, Park SB, Vucic S, Carey KA, Turner BJ, Gillingwater TH (2017). Emerging therapies and challenges in spinal muscular atrophy. Ann Neurol.

[3] Wadman R, Wijngaarde C, Stam M, Bartels B, Otto L, Lemmink H (2018). Muscle strength and motor function throughout life in a cross-sectional cohort of 180 patients with spinal muscular atrophy types 1c–4. Eur J Neurol.

[4] Febrer A, Rodriguez N, Alias L, Tizzano E (2010). Measurement of muscle strength with a handheld dynamometer in patients with chronic spinal muscular atrophy. J Rehabil Med.

[5] Deymeer F, Serdaroglu P, Parman Y, Poda M (2008). Natural history of sma iiib muscle strength decreases in a predictable sequence and magnitude. Neurology..

[6] Finkel RS, Mercuri E, Meyer OH, Simonds AK, Schroth MK, Graham RJ (2018). Diagnosis and management of spinal muscular atrophy: part 2: pulmonary and acute care; medications, supplements and immunizations; other organ systems; and ethics. Neuromuscul Disord.

[7] Mercuri E, Finkel RS, Muntoni F, Wirth B, Montes J, Main M (2018). Diagnosis and management of spinal muscular atrophy: part 1: recommendations for diagnosis, rehabilitation, orthopedic and nutritional care. Neuromuscul Disord.

[8] Wang CH, Finkel RS, Bertini ES, Schroth M, Simonds A, Wong B (2007). Consensus statement for standard of care in spinal muscular atrophy. J Child Neurol.

[9] Farrar MA, Carey KA, Paguinto SG, Chambers G, Kasparian NA (2018). Financial, opportunity and psychosocial costs of spinal muscular atrophy: an exploratory qualitative analysis of australian carer perspectives. BMJ Open.

[10] Farrar MA, Teoh HL, Carey KA, Cairns A, Forbes R, Herbert K (2018). Nusinersen for sma: expanded access programme. J Neurol Neurosurg Psychiatry.

[11] Michelson D, Ciafaloni E, Ashwal S, Lewis E, Narayanaswami P, Oskoui M, et al. Evidence in focus: Nusinersen use in spinal muscular atrophy: report of the guideline development, dissemination, and implementation subcommittee of the American Academy of Neurology. Neurology. 2018. 10.1212/WNL.0000000000006502. 10.1212/WNL.0000000000006502 30315070

[12] Verhaart I, Robertson A, Leary R, McMacken G, König K, Kirschner J (2017). A multi-source approach to determine sma incidence and research ready population. J Neurol.

[13] López-Bastida J, Peña-Longobardo LM, Aranda-Reneo I, Tizzano E, Sefton M, Oliva-Moreno J (2017). Social/economic costs and health-related quality of life in patients with spinal muscular atrophy (sma) in Spain. Orphanet J Rare Dis.

[14] Rouault F, Christie-Brown V, Broekgaarden R, Gusset N, Henderson D, Marczuk P (2017). Disease impact on general well-being and therapeutic expectations of European type II and type III spinal muscular atrophy patients. Neuromuscul Disord.

[15] Klug C, Schreiber-Katz O, Thiele S, Schorling E, Zowe J, Reilich P (2016). Disease burden of spinal muscular atrophy in Germany. Orphanet J Rare Dis..

[16] Brett J, Staniszewska S, Mockford C, Herron-Marx S, Hughes J, Tysall C (2014). Mapping the impact of patient and public involvement on health and social care research: a systematic review. Health Expect.

[17] Ocloo J, Matthews R. From tokenism to empowerment: progressing patient and public involvement in healthcare improvement. BMJ Qual Saf. 2016. 10.1136/bmjqs-2015-004839. 10.1136/bmjqs-2015-004839 PMC4975844 26993640

[18] Mockford C, Staniszewska S, Griffiths F, Herron-Marx S (2011). The impact of patient and public involvement on UK NHS health care: a systematic review. Int J Qual Health Care.

[19] Crawford MJ, Rutter D, Manley C, Weaver T, Bhui K, Fulop N (2002). Systematic review of involving patients in the planning and development of health care. BMJ..

[20] Corbin J, Strauss A. Basics of Qualitative Research. 3rd Edition. Thousand Oaks: Sage Publications Ltd; 2015.

[21] Miles MB, Huberman AM, Saldaña J (2014). Qualitative data analysis: A methods sourcebook.

[22] Ho HM, Tseng YH, Hsin YM, Chou FH, Lin WT (2016). Living with illness and self-transcendence: the lived experience of patients with spinal muscular atrophy. J Adv Nurs.

[23] Lamb C, Peden A (2008). Understanding the experience of living with spinal muscular atrophy: a qualitative description. J Neurosci Nurs.

[24] Jeppesen J, Madsen A, Marquardt J, Rahbek J (2010). Living and ageing with spinal muscular atrophy type 2: observations among an unexplored patient population. Dev Neurorehabil.

[25] Hunter M, Heatwole C, Luebbe E, Johnson NE (2016). What matters most: a perspective from adult spinal muscular atrophy patients. J Neuromuscul Dis.

[26] Qian Y, McGraw S, Henne J, Jarecki J, Hobby K, Yeh W (2015). Understanding the experiences and needs of individuals with spinal muscular atrophy and their parents: a qualitative study. BMC Neurol.

[27] Kruitwagen-Van Reenen ET, Wadman RI, Visser-Meily JM, van den Berg LH, Schröder C, van der Pol WL (2016). Correlates of health related quality of life in adult patients with spinal muscular atrophy. Muscle Nerve.

[28] Mongiovi P, Dilek N, Garland C, Hunter M, Kissel JT, Luebbe E (2018). Patient reported impact of symptoms in spinal muscular atrophy (PRISM-SMA). Neurology..

[29] Molster C, Urwin D, Di Pietro L, Fookes M, Petrie D, van der Laan S (2016). Survey of healthcare experiences of Australian adults living with rare diseases. Orphanet J Rare Dis..

[30] Ritchie J, Lewis J, McNaughton Nicholls C, Ormston R. Qualitative research practice: a guide for social science students and researchers. London: Sage Publications Ltd; 2014.

[31] Marshall MN (1996). Sampling for qualitative research. J Fam Pract.

[32] Morse JM (1995). The significance of saturation.

[33] Tong A, Sainsbury P, Craig J (2007). Consolidated criteria for reporting qualitative research (COREQ): a 32-item checklist for interviews and focus groups. Int J Qual Health Care.

[34] Budych K, Helms TM, Schultz C (2012). How do patients with rare diseases experience the medical encounter? Exploring role behavior and its impact on patient–physician interaction. Health Policy.

[35] Griggs RC, Batshaw M, Dunkle M, Gopal-Srivastava R, Kaye E, Krischer J (2009). Clinical research for rare disease: opportunities, challenges, and solutions. Mol Genet Metab.

[36] Schieppati A, Henter J-I, Daina E, Aperia A (2008). Why rare diseases are an important medical and social issue. Lancet..

[37] Sharma A, Jacob A, Tandon M, Kumar D (2010). Orphan drug: Development trends and strategies. J Pharm Bioallied Sci.

[38] Oliveira P, Zejnilovic L, Canhão H, von Hippel E (2015). Innovation by patients with rare diseases and chronic needs. Orphanet J Rare Dis..

[39] Kasparian NA, Rutstein A, Sansom-Daly UM, Mireskandari S, Tyler J, Duffy J (2015). Through the looking glass: an exploratory study of the lived experiences and unmet needs of families affected by Von Hippel–Lindau disease. Eur J Hum Genet.

[40] van Haastregt JCM, de Witte LP, Terpstra SJ, Diederiks JPM, van der Horst FGEM, de Geus CA (1994). Membership of a patients’ association and well-being a study into the relationship between membership of a patients’ association, fellow-patient contact, information received, and psychosocial well-being of people with a neuromuscular disease. Patient Educ Couns.

[41] Green EC, Murphy E. Health belief model. In W. Cockeringham, R. Dingwall, & S. Quah, (Eds.), The Wiley Blackwell encyclopedia of health, illness, behavior, and society. Hoboken: Wiley. 2014;2:766–769.

[42] Hochbaum G, Rosenstock I, Kegels S (1952). Health belief model.

[43] Galdas PM, Cheater F, Marshall P (2005). Men and health help-seeking behaviour: literature review. J Adv Nurs.

[44] Andrade LH, Alonso J, Mneimneh Z, Wells JE, Al-Hamzawi A, Borges G (2013). Barriers to mental health treatment: results from the WHO world mental health surveys. Psychol Med.

[45] Hale S, Grogan S, Willott S (2010). Male GPs’ views on men seeking medical help: a qualitative study. Br J Health Psychol.

[46] McGraw S, Qian Y, Henne J, Jarecki J, Hobby K, Yeh W-S (2017). A qualitative study of perceptions of meaningful change in spinal muscular atrophy. BMC Neurol.

[47] Doyle C, Lennox L, Bell D (2013). A systematic review of evidence on the links between patient experience and clinical safety and effectiveness. BMJ Open.

[48] Springham N, Robert G (2015). Experience based co-design reduces formal complaints on an acute mental health ward. BMJ Open Qual.

[49] Sharma AE, Knox M, Mleczko VL, Olayiwola JN (2017). The impact of patient advisors on healthcare outcomes: a systematic review. BMC Health Serv Res.

[50] Robert G, Cornwell J, Locock L, Purushotham A, Sturmey G, Gager M (2015). Patients and staff as codesigners of healthcare services. BMJ..

[51] Piper D, Iedema R, Gray J, Verma R, Holmes L, Manning N (2012). Utilizing experience-based co-design to improve the experience of patients accessing emergency departments in new South Wales public hospitals: an evaluation study. Health Serv Manag Res.

[52] Crowley R, Wolfe I, Lock K, McKee M (2011). Improving the transition between paediatric and adult healthcare: a systematic review. Arch Dis Child.

[53] Gholap N, Pillai M, Virmani S, Lee JD, James D, Morrissey J (2006). The alphabet strategy and standards of care in young adults with type 1 diabetes. Br J Diabetes Vasc Dis.

[54] Cadario F, Prodam F, Bellone S, Trada M, Binotti M, Trada M (2009). Transition process of patients with type 1 diabetes (T1DM) from paediatric to the adult health care service: a hospital-based approach. Clin Endocrinol.

[55] Vidal M, Jansa M, Anguita C, Torres M, Gimenez M, Esmatjes E (2004). Impact of a special therapeutic education programme in patients transferred from a paediatric to an adult diabetes unit. Eur Diabetes Nurs.

[56] Blum R, Hirsch D, Kastner T, Quint R (2002). A consensus statement on health care transitions for young adults with special health care needs. Pediatrics..

[57] Kruitwagen-van Reenen ET, van der Pol L, Schröder C, Wadman RI, van den Berg LH, Visser-Meily JM (2018). Social participation of adult patients with spinal muscular atrophy: frequency, restrictions, satisfaction and correlates. Muscle Nerve.

[58] Kenzik KM (2018). Health care use during cancer survivorship: review of 5 years of evidence. Cancer..

[59] Almeida SN, Elliott R, Silva ER, Sales CM (2018). Fear of cancer recurrence: a qualitative systematic review and meta-synthesis of patients’ experiences. Clin Psychol Rev.

[60] McEvoy P, Barnes P (2007). Using the chronic care model to tackle depression among older adults who have long-term physical conditions. J Psychiatr Ment Health Nurs.

[61] Dieng M, Butow PN, Costa DS, Morton RL, Menzies SW, Mireskandari S (2016). Psychoeducational intervention to reduce fear of cancer recurrence in people at high risk of developing another primary melanoma: results of a randomized controlled trial. J Clin Oncol.

[62] Dieng M, Morton RL, DSJ C, Butow P, Menzies S, Lo S (2018). Sustained long-term benefits of a psycho-educational intervention targeting fear of cancer recurrence in people at high risk of developing another melanoma: a randomised controlled trial. J Clin Oncol.

